# Seven cardinal questions for the patient with ear, nose or throat complaints: Review

**DOI:** 10.1097/MD.0000000000031852

**Published:** 2022-12-16

**Authors:** Sapideh Gilani

**Affiliations:** a Division of Otolaryngology, Department of Surgery, University of California San Diego, San Diego, CA, USA.

**Keywords:** adult, anatomy, clinical, consensus, ear, ENT, evaluation, evidence-based, family, general, guideline, head, history, medical, medicine, neck, nose, otolaryngology, outcome, physical, practice, practice, sinus, surgery, throat

## Abstract

The incidence of otolaryngological problems in general medicine practice is highly seasonal and approaches 25% in some months of the year. Accurate diagnosis in the otolaryngology office often requires the use of binocular microscopy, nasal endoscopy, and flexible laryngoscopy, none of which are available in a general medicine office. Therefore, history taking and physical examination techniques available in the non-otolaryngology office are of utmost importance. Using evidence-based history taking for ear, nose, and throat (ENT) problems facilitates dealing with patients who present with otolaryngologic complaints. In this paper, I present 7 cardinal questions to be asked when taking the history of a patient with ENT complaints.

## 1. Introduction

Previous systematic reviews have concluded that most medical schools do not require otolaryngology instruction^[1]^ despite the high incidence of ENT problems in the general patient population. In an ambulatory general medicine practice 10% of patients on average present with otolaryngologic chief complaints, and during some months of the year this number may increase to 25% of pediatric and adult patients.^[2]^

For medical practitioners who have never had exposure to otolaryngology as students, making sense of the otolaryngology patient constellation of symptoms may be daunting. Several clinical practice guidelines have been developed to guide physicians in the care of common otolaryngology problems in adults and children.^[3–10]^ Using the recommendations from these guidelines, I suggest evidence-based cardinal questions to be used when taking the history of an adult ear,^[5,7,9,11]^ nose^[3,4,12–15]^ and throat patients.^[16,17]^ To my knowledge, this is the first paper to present essential and cardinal questions to be used in history taking of patients with otolaryngologic complaints.

## 2. Materials and methods

Basic history taking includes asking “OLD CARTS,” an acronym referring to onset, location, duration, character, aggravating and associated factors, relieving factors, timing, and severity of symptoms. In addition, medical history, past surgical history, medications, allergies, and social history, including smoking, alcohol use, and occupation, are reviewed, as is the case for all patients. The patients’ vitals are reviewed. ENT problems are addressed using 7 cardinal questions for each problem.Table 1 If a patient presents with a problem in more than 1 otolaryngologic area, then all 7 cardinal questions are repeated for that area. The severity of the complaint is assessed on a 10 point scale with 0 as no problem and 10 as the worst imaginable problem. University of California San Diego institutional review board deems this review exempt.

**Table 1:** Seven cardinal questions to ask of the patient with ENT complaints.

Anatomic area	Seven cardinal questions	
	Evidence based	Confirmation
Ear	Hearing loss	Ear surgery
	Tinnitus	Noise/chemical/mechanical trauma
	Vertigo	Family history of hearing loss
	Ear fullness level out of 10	
Nose	Nasal obstruction	Nose surgery
	Sense of smell	Nose trauma
	Discolored nasal discharge Facial pain level out of 10	Obstructive sleep apnea
Throat	Globus	Smoking history
	Dysphagia	Alcohol history
	Reflux symptoms Odynophagia level out of 10	Proton pump inhibitor or histamine 2 receptor blocker use

ENT = ear, nose or throat.

## 3. Discussion

In the UK a systematic review of undergraduate medical students’ opinion of caring for patients with ENT problems found their preparedness to be low.^[18]^ Similar conclusions have been drawn in other parts of the world regarding low medical student preparedness for otolaryngology patients.^[19–21]^ Despite the sense of unpreparedness to care for otolaryngology patients, a study of UK medical schools found that mandatory ENT rotations were only provided to 53% of medical students^[22]^ and the mean duration of the rotation was only 8 days.^[22]^ Similarly a survey of medical schools within the US found that 89% offered otolaryngology as an elective rotation, and only 7% of US medical schools had a mandatory otolaryngology clinical rotation, with an average of only 12 students per school participating yearly across the average of all medical schools including schools offering electives and having mandatory otolaryngology rotations.^[23]^ At University of California San Diego, less than one third of students rotate on otolaryngology, and the rotation is not mandatory. Previous studies have emphasized various methods of teaching otolaryngology to medical students, including problem-based,^[21,24]^ simulation and e-learning,^[25]^ head and neck exam^[20,26,27]^ and anatomy lectures.^[20,28]^ Previous studies have found that having a student in the clinic does not affect appointment times and patient satisfaction is neither increased nor decreased by medical student participation, while student satisfaction is increased.^[29]^ However, many otolaryngology programs in academic settings have not increased the cohort of students exposed to otolaryngology. This lack of familiarity with the field of otolaryngology leaves many who choose fields outside otolaryngology overwhelmed with the patient with 1 or more otolaryngologic complaints.

Using evidence-based cardinal questions when taking the history of an adult ear,^[5,7,9,11]^ nose^[3,4,12–15]^ and throat patient.^[16,17]^ has not previously been described in the literature.

Utilizing the relevant clinical practice guidelines to ask relevant questions of the ENT patient during history taking greatly facilitates the work-up of patients with otolaryngologic chief complaints. For nose patients, questions on the sinonasal outcome test-22 (SNOT-22)^[30]^ and throat patients asking all questions on the voice handicap index (VHI)^[31,32]^ can be used as an intake form when evaluating a patient with an otolaryngology complaint; however, this takes time and adds to the complexity of the visit process. Using the 7 evidence-based cardinal questions when taking the history of a patient with an otolaryngology complaint is a much shorter and easier process. Asking about the pain level for each ENT complaint is essential, as many patients have minimal complaints but are mostly concerned about having a deadly disease in the area of concern.

One drawback of using the 7 cardinal questions is that not all possible questions are included for every single otolaryngologic diagnosis; however, the benefit of cardinal questions is the simplicity, consistency, and having a framework on which to build otolaryngology knowledge. Additionally, the structured approach of asking 7 cardinal questions permits evolutionary improvement over time, building on a documented foundation for future ambulatory ENT patients.^[23,24,27,33]^

## 4. Conclusion

Using the 7 cardinal questions when taking ENT history, is a useful tool for non-otolaryngologists and serves as a framework for adding future otolaryngologic knowledge and facilitating caring for patients with an otolaryngology complaint.
